# Toward production of jet fuel functionality in oilseeds: identification of FatB acyl-acyl carrier protein thioesterases and evaluation of combinatorial expression strategies in *Camelina* seeds

**DOI:** 10.1093/jxb/erv225

**Published:** 2015-05-11

**Authors:** Hae Jin Kim, Jillian E. Silva, Hieu Sy Vu, Keithanne Mockaitis, Jeong-Won Nam, Edgar B. Cahoon

**Affiliations:** ^1^Department of Biochemistry and Center for Plant Science Innovation, University of Nebraska-Lincoln, Lincoln, NE 68588, USA; ^2^Department of Biology, and Center for Genomics and Bioinformatics, Indiana University, Bloomington, IN 47405, USA; ^3^Donald Danforth Plant Science Center, Saint Louis, MO 63132, USA

**Keywords:** *Camelina*, *Cuphea*, FatB acyl-ACP thioesterase, jet fuel oilseed, medium-chain fatty acid.

## Abstract

Seeds of members of the genus *Cuphea* accumulate medium-chain fatty acids (MCFAs; 8:0–14:0). MCFA- and palmitic acid- (16:0) rich vegetable oils have received attention for jet fuel production, given their similarity in chain length to Jet A fuel hydrocarbons. Studies were conducted to test genes, including those from *Cuphea*, for their ability to confer jet fuel-type fatty acid accumulation in seed oil of the emerging biofuel crop *Camelina sativa*. Transcriptomes from *Cuphea viscosissima* and *Cuphea pulcherrima* developing seeds that accumulate >90% of C8 and C10 fatty acids revealed three *FatB* cDNAs (*CpuFatB3*, *CvFatB1*, and *CpuFatB4*) expressed predominantly in seeds and structurally divergent from typical FatB thioesterases that release 16:0 from acyl carrier protein (ACP). Expression of *CpuFatB3* and *CvFatB1* resulted in *Camelina* oil with capric acid (10:0), and *CpuFatB4* expression conferred myristic acid (14:0) production and increased 16:0. Co-expression of combinations of previously characterized *Cuphea* and California bay FatBs produced *Camelina* oils with mixtures of C8–C16 fatty acids, but amounts of each fatty acid were less than obtained by expression of individual *FatB* cDNAs. Increases in lauric acid (12:0) and 14:0, but not 10:0, in *Camelina* oil and at the *sn*-2 position of triacylglycerols resulted from inclusion of a coconut lysophosphatidic acid acyltransferase specialized for MCFAs. RNA interference (RNAi) suppression of *Camelina* β-ketoacyl-ACP synthase II, however, reduced 12:0 in seeds expressing a 12:0-ACP-specific FatB. *Camelina* lines presented here provide platforms for additional metabolic engineering targeting fatty acid synthase and specialized acyltransferases for achieving oils with high levels of jet fuel-type fatty acids.

## Highlight

Transcriptomic data mining and combinatorial gene stacking was used for metabolic engineering of *Camelina* to produce oils containing medium-chain length fatty acids that mimic jet fuel hydrocarbon components.

## Introduction

Medium-chain fatty acids (MCFAs), including caprylic acid (8:0), capric acid (10:0), lauric acid (12:0), and myristic acid (14:0), are important for industrial production of detergents, soaps, cosmetics, surfactants, and lubricants (Knaut and Richtler, 1985; Dyer *et al.*, 2008). These fatty acids, along with palmitic acid (16:0), also have potential for use as feedstocks for the hydrocarbon component of Jet A and Jet A-1 fuels, which are composed primarily of C8–C16 alkanes and aromatic hydrocarbons (Hemighaus *et al.*, 2006; Kallio *et al.*, 2014). The main commercial sources of plant-derived MCFAs are oils from tropical plants including palm kernel (*Elaeis guineensis* Jacq.) and coconut (*Cocos nucifers* L.), which are enriched in lauric acid (46–52 mol% of total fatty acids) and myristic acid (16–19 mol% of total fatty acids). Among the few temperate sources of MCFAs are seeds from members of the *Cuphea* genus, some of which produce >90% of a single MCFA (Graham and Kleiman, 1992; Graham, 1998). Examples of *Cuphea* species with seeds that accumulate high levels of MCFAs include *Cuphea viscosissima*, with seeds that contain ~25 mol% 8:0 and ~64 mol% 10:0, and *Cuphea pulcherrima*, with seeds that contain ~95 mol% 8:0. *Cuphea* oil showed favourable fuel properties for biodiesel in functionality testing due to its high content of MCFAs (Geller *et al.*, 1999; Knothe *et al.*, 2009; Lovestead *et al.*, 2010; Knothe, 2014). Even though many *Cuphea* species have been characterized as the potential oil crop for MCFAs, non-desired traits such as indeterminate flowering, seed shattering, seed dormancy, viscid and glandular trichomes in vegetative tissues and flowers, and open pollination have limited attempts to domesticate *Cuphea* species for agronomic production (Olejniczak, 2011). Therefore, *Cuphea* species have been considered as valuable genetic resources to isolate genes that encode specialized biosynthetic enzymes for transgenic production of MCFAs in established oilseed crops (Dehesh *et al.*, 1996a, b; Leonard *et al.*, 1997, 1998; Slabaugh *et al.*, 1998; Filichkin *et al.*, 2006).

The synthesis of MCFAs is a variation on typical *de novo* fatty acid synthesis that occurs in plants that generates primarily C16 and C18 fatty acids. *De novo* fatty acid biosynthesis occurs in the plastids in plants and is initiated by the condensation of acetyl-coenzyme A (CoA) and malonyl-acyl carrier protein (ACP) by the β-ketoacyl-ACP synthase III (KASIII) to produce a four-carbon β-ketoacyl-ACP. Fatty acids are elongated by sequential condensation of two carbon units from malonyl-ACP by the co-operation of enzymes of fatty acid synthase (FAS). Following its synthesis, 16:0-ACP can be further elongated to 18:0-ACP by KASII or is hydrolysed by acyl-ACP thioesterase to generate free palmitic acid that is exported from plastids. Hydrolysis of 16:0-ACP is catalysed primarily by FatB thioesterases, whereas hydrolysis of ACP esters of the C18 fatty acids, stearic acid (18:0) and oleic acid (18:1), is catalysed principally by FatA thioesterases. As such, acyl-ACP thioesterases are major determinants of carbon chain lengths of fatty acid (Li-Beisson *et al.*, 2013). Divergent FatB enzymes with substrate specificities for saturated fatty acids with chain lengths less than C16 are responsible for the synthesis of MCFAs in seeds of *Cuphea* species as well as species such as California bay (*Umbellularia californica*). These divergent FatBs have been used to confer MCFA production to *Brasicca napus* and *Arabidopsis thaliana* by transgenic expression (Pollard *et al.*, 1991; Voelker *et al.*, 1992; Jones *et al.*, 1995; Dehesh *et al.*, 1996b; Eccleston *et al.*, 1996; Tjellström *et al.*, 2013). An alternative fate to release of 16:0 from ACP by typical FatB thioesterase is elongation of 16:0-ACP to 18:0-ACP, which is initiated by KASII activity. Mutagenesis or RNA interference (RNAi) suppression of KASII genes has been shown to be an effective way of generating 16:0-rich oils, which could potentially contribute to bio-based jet fuel (Pidkowich *et al.*, 2007).

Jet A fuel-type fatty acids (MCFAs and 16:0) released from the plastid of oilseeds must be esterified onto glycerol backbones in the endoplasmic reticulum (ER) for sequestration in triacylglycerol (TAG). Key to achieving high levels of Jet A fuel-type fatty acid accumulation in TAG is the acyltransferases glycerol-3-phosphate acyltransferase (GPAT), lysophosphatidic acid acyltransferase (LPAT), and diacylglycerol acyltransferase (DGAT) that catalyse the sequential addition of CoA esters of MCFAs and 16:0 to the TAG glycerol backbone (Thelen and Ohlrogge, 2002; Cahoon *et al.*, 2007; Dyer *et al.*, 2008; Lu *et al.*, 2011). To obtain levels of MCFA and 16:0 accumulation at levels found in seed TAG of many *Cuphea* species, these fatty acids must be introduced at all stereospecific positions of TAG (Knutzon *et al.*, 1999; Dehesh, 2001; Roscoe, 2005; Burgal *et al.*, 2008; Snyder *et al.*, 2009). Limiting this in most oilseed crops is the strict substrate specificity of LPAT for unsaturated acyl-CoAs, such as oleoyl (18:1)-CoA (Kim *et al.*, 2005; Nlandu Mputu *et al.*, 2009). As such, a major target for engineering high levels of MCFA and 16:0 accumulation in engineered oilseeds is the identification of LPATs and possibly other acyltransferases with specificity for CoA esters of these saturated fatty acids.

In this report, new divergent *FatB* genes have been identified from transcriptomes of developing seeds from *C. viscosissima* and *C. pulcherrima*. The emerging oilseed crop [*Camelina sativa* (L.) Crantz] was used to characterize *FatB* genes, with the goal of generating Jet A and Jet A-1 fuel-type fatty acid compositions in vegetable oils. *Camelina* is a member of the Brassicaceae family and is currently being developed as a non-food oilseed for biofuel, industrial, and high-value specialty oil traits (Iskandarov *et al.*, 2014). *Camelina* is especially attractive as a biotechnological crop because it is readily transformed by floral *Agrobacterium* infiltration (Lu and Kang, 2008; Liu *et al.*, 2012). In addition, *Camelina* is productive in the climate of the North American Great Plains and Pacific Northwest, characterized by limited rainfall and marginal soil fertility. These properties together with its short 100–120 d life cycle have made *Camelina* attractive for production in fallow seasons and in double-cropping systems (Iskandarov *et al.*, 2014). *Camelina* seeds are also oil rich (30–40% of seed weight), but the oil contains high levels of polyunsaturated fatty acids, which reduce the biofuel quality of *Camelina* oil because of their oxidative instability (Johnson *et al.*, 2011; Campbell *et al.*, 2013; Iskandarov *et al.*, 2014). Genetic improvement of *Camelina* for biofuel and industrial and specialty oil traits has been facilitated in part by the recent release of a genome sequence and seed transcriptomes that include compilation of oil and seed storage gene databases (Nguyen *et al.*, 2013, 2014; Kagale *et al.*, 2014).

In these studies, the newly identified *Cuphea FatB* genes as well as previously reported *FatB* genes from several *Cuphea* species and California bay were expressed individually and in combination in *Camelina* seeds to generate oils with a range of MCFA compositions. Co-expression studies of divergent *FatB* genes with a specialized LPAT from coconut were also conducted to evaluate the effectiveness of this strategy for increasing MCFA accumulation in *Camelina* seed TAG. RNAi-mediated down-regulation of *Camelina KASII* was also tested as a means of increasing accumulation of MCFAs as well as 16:0 in seeds from *Camelina* lines engineered for overexpression of functionally divergent FatBs.

## Materials and methods

### Plant material, growth, and transformation conditions

*Camelina sativa* (variety Suneson) seeds were sown into 81cm^2^ plastic pots containing Fafard Germination Mix media (Hummert International, Saint Louis, MO, USA). Ambient light was supplemented in greenhouses with a combination of metal halide and high pressure sodium lights with a 14h daylength. Day temperatures ranged from 24 ºC to 26 ºC, and night temperatures ranged from 18 ºC to 20 ºC. When outdoor temperatures were >29 ºC, supplemental lights were shut off to reduce the need for extra cooling. *Agrobacterium tumefaciens* cells (strain C58C1) with the binary vectors containing FatB thioesterase cDNAs were transformed by electroporation. *Camelina* plants were transformed by floral dip/vacuum infiltration, and DsRed (*Discosoma* sp. red fluorescent protein) was used as a visual selection marker (Lu and Kang, 2008). Segregation patterns of T_2_ seed were used to determine lines containing a single T-DNA insertion, and homozygous lines were subsequently isolated as the T_3_ progeny of single-insert plants having 100% of seed showing red fluorescence.

### Binary vectors

Previously reported binary vectors were used for expression of *CpuFatB3*, *CpFatB2*, *UcFatB1*, or *ChFatB2* in *Camelina* seeds (Tjellström *et al.*, 2013). Full-length cDNAs for *CpuFatB1* and *CpuFatB4* genes from *C. pulcherrima* developing seed and *CvFatB1* and *CvFatB3* from *C. viscosissima* developing seed cDNA were amplified by PCR using the oligonucleotide primers with added *Eco*RI and *Xba*I restriction sites. The *Eco*RI- and *Xba*I-digested fragments containing the genes were inserted into the corresponding sites of the pBinGlyRed3 binary expression vector which contains the strong seed-specific soybean glycinin-1 promoter and a DsRed marker gene (Zhang *et al.*, 2013). The primer sequences are provided in Supplementary Table S1 available at *JXB* online. Primers were designed to amplify the open reading frames (ORFs) of those from developing seeds, and product sequences were confirmed by sequencing.

### RNA isolation and cDNA synthesis for RT-PCR

Total RNA was isolated from *Cuphea* roots, stems, leaves, flowers, and developing seeds using an RNeasy Plant Mini Kit (Qiagen, Valencia, CA, USA) with methods slightly modified from those described previously (Chang *et al.*, 1993). A pre-heated 10ml of extraction buffer [2% (w/v) cetyltrimethylammonium bromide (CTAB), 2% (w/v) polyvinylpyrrolidone (PVP), 2M NaCl, 100mM TRIS-HCl pH 8.0, 25mM EDTA pH 8.0, and 0.05% (w/v) spermidine] was added to the sample (200–300mg) ground in liquid nitrogen, mixed, and incubated at 65 °C for 10min. An equal volume of chloroform was added, mixed, and centrifuged at 15 000 *g* for 10min at 4 °C. One-third volume of 8M LiCl was added to the supernatant and incubated on ice overnight. RNA was collected by centrifugation at 15 000 *g* for 1h at 4 °C. The pellet was resuspended in 500 μl of RLT buffer, and DNase I treatment was carried out according to the manufacturer’s protocol. First-strand cDNA was synthesized from 2 μg of total RNA using a RevertAid First Strand cDNA Synthesis Kit (ThermoFisher Scientific, Waltham, MA, USA) with an oligo(dT) primer.

### Fatty acid analysis of seed oils

Fatty acid methyl esters (FAMEs) were generated by grinding 10mg of dry seeds in 2ml of 2.5% H_2_SO_4_ (v/v) in methanol including 900 μg of tri 17:0-TAG (Nu-Chek Prep, Elysian, MN, USA) in toluene (10mg ml^–1^) as an internal standard and heated for 45min at 90 °C in tightly capped tubes. Following cooling, 1.5ml of water and 1.5ml of hexane were added to the tubes and mixed. The organic phase was transferred to autosampler vials and analysed on an Agilent Technologies 7890A gas chromatograph fitted with a 30 m length×0.25mm inner diameter HP-INNOWax column (Agilent, Santa Clara, CA, USA) using H_2_ carrier gas. The gas chromatograph was programmed for an initial temperature of 90 ºC (1min hold) followed by an increase of 30 ºC min^–1^ to 235 ºC and maintained for a further 5min. Detection was achieved using flame ionization.

### Analysis of the *sn*-2 position of TAG

Total neutral lipid was extracted from seeds using a modification of the Bligh–Dyer method (Bligh and Dyer, 1959). A 30mg aliquot of *Camelina* seeds was ground in 3ml of methanol:chloroform (2:1 v/v) with 2.7mg of C17-TAG as an internal standard. Homogenized samples were incubated for 30min to 1h at room temperature with agitation, and lipids were partitioned and extracted as described (Cahoon *et al.*, 2006). Total lipids were re-dissolved in 1ml of heptane. To purify TAG, a 3ml Supelco Supel Clean LC-Si SPE column (Sigma-Aldrich, Saint Louis, MO, USA) was used. The column was equilibrated with 5ml of heptane. The total lipid sample in heptane was added to the column and drained into the column bed. To remove extra wax ester, 1.5ml of heptane:diethyl ether (95:5, v/v) was added to the column and this fraction was discarded. Then, 5ml of heptane:diethyl ether (80:20, v/v) was added and this fraction was collected as TAG. Analysis of TAG *sn*-2 fatty acids was conducted using lipase digestion as described (Cahoon *et al.*, 2006).

### Phylogenetic tree of *Cuphea* FatB amino acid sequence clusters

A phylogenic tree was generated by MEGA6 software, using the minimum-evolution method with 1000 bootstrap replications (Tamura *et al.*, 2013). Multiple sequence alignments were produced using ClustalW (http://www.genome.jp/tools-bin/clustalw). The MSF-formatted alignment was then analysed using the Genedoc sequence analysis program (Nicholas *et al.*, 1997).

### 454 transcriptome sequencing and transcriptome assembly

Total RNA was extracted from developing seeds (between 14 d and 18 d after pollination) of *C. pulcherrima* and *C. viscosissima* using a method as described (Suzuki *et al.*, 2004) without 8M LiCl treatment. mRNAs were purified from ~1mg of total RNA by two passes through oligo(dT)–cellulose columns by use of the Illustra mRNA purification kit (GE Healthcare, Pittsburgh, PA, USA). A sequencing library optimized for Roche/454 GS FLX Titanium sequencing was prepared from oligo(dT)-enriched mRNA according to custom protocols used previously (Nguyen *et al.*, 2013). To reduce the number of high copy transcripts, amplified dsDNA library intermediates were partially normalized using Trimmer Direct (Evrogen) protocols. Emulsion PCR and sequencing were performed according to the manufacturer (Roche/454 Life Sciences). High quality sequence reads were trimmed (https://sourceforge.net/projects/estclean/) and assembled using Newbler v2.0.

### Acyl-ACP analysis

Acyl-ACPs were extracted as previously described (Bates *et al.*, 2014). The enriched acyl-ACP samples were treated with Asp-N endoproteinase (Sigma-Aldrich) at a 1:50 protein ratio, and incubated at 37 °C for 2h. Methanol was added to a final concentration of 50% after enzyme digestion. Mass spectrometry analyses were conducted using a 4000 QTRAP (Applied Biosystems) LC-MS/MS as described (Bates *et al.*, 2014).

## Results

### Fatty acid profile of *Cuphea* seeds and leaves

**Fig. 1.**
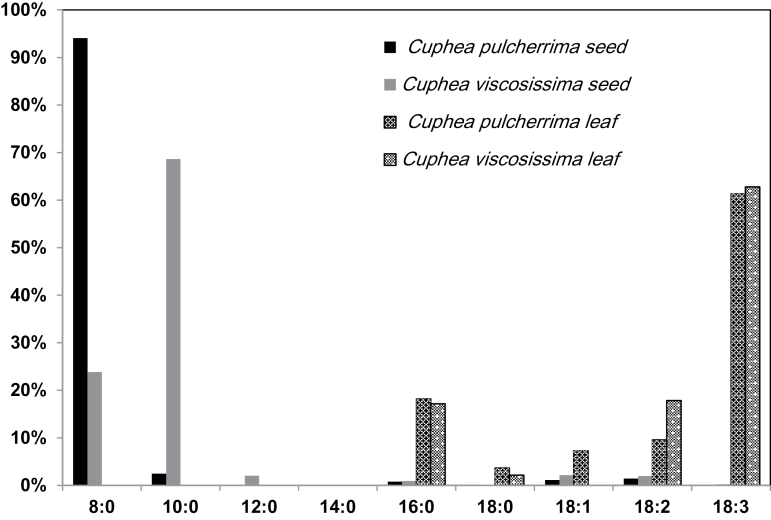
Fatty acid composition of seeds and leaves in *Cuphea pulcherrima* and *Cuphea viscosissima*. Fatty acids were extracted from leaves and seeds of *C. pulcherrima* and *C. viscosissima* and analysed using gas chromatography. Values are the means ±SD from five biological replicates.

Seeds of *C. pulcherrima* and *C. viscosissima* accumulate 94 mol% of 8:0, and 17 mol% of 8:0 and 70 mol% of 10:0 fatty acids, respectively (Graham and Kleiman, 1992; Phippen *et al.*, 2006). To confirm the fatty acid profile of *Cuphea* seeds and to compare their profile with other tissues, the fatty acid composition was analysed in mature dried seeds and leaves. Seeds and leaves of *C. pulcherrima* and *C. viscosissima* had very different fatty acid compositions (Fig. 1). As previously reported, 8:0 accounted for nearly 95 mol% of fatty acids in *C. pulcherrima* seeds, while the leaves contained primarily 16:0 and large amounts of unsaturated long fatty acids, including 18:3, which made up 61 mol% of the total fatty acids. Similar results were observed in *C. viscosissima* (Fig. 1). Nearly 90 mol% of the fatty acids in *C. viscosissima* seed consisted of 8:0 and 10:0, but leaves were enriched in 18:3 in amounts similar to those in *C. pulcherrima* leaves. This result confirmed that seeds of *C. pulcherrima* and *C. viscosissima* are excellent genetic resources for caprylic acid (8:0) and capric acid (10:0).

Transcriptomic analyses using 454 pyrosequencing was conducted on normalized cDNAs from *C. pulcherrima* and *C. viscosissima* developing seeds to identify specialized FatBs and metabolic enzymes (e.g. acyltransferases) associated with MCFA synthesis and accumulation. From the transcriptomic analyses, four *FatB* cDNAs, designated *CpuFatB1*, *CpuFatB2*, *CpuFatB3*, and *CpuFatB4*, were identified in *C. pulcherrima*. Of these, only CpuFatB1 activity was tested previously by expression in *Camelina* seeds, and shown to be a typical FatB that generated seed oils enriched in palmitic acid (16:0) (Horn *et al.*, 2013). In addition, three *FatB* cDNAs, designated *CvFatB1*, *CvFatB2*, and *CvFatB3*, were identified in 454 transcriptomic analyses of *C. viscosissima* genes. These cDNAs were nearly identical to *C. viscosissima FatB* cDNAs previously deposited in the National Center for Biotechnology Information (NCBI) database. Notably, *CvFatB3* identified in the transcriptomic analyses differed from the analogous sequence in NCBI by two nucleotides that resulted in one amino acid difference (A instead of V), and the gain of a stop codon resulting in a polypeptide of 388 amino acids rather than 412 amino acids as found in the existing NCBI accession.

### Analysis of the spatial expression of FatB transcripts in *C. pulcherrima* and *C. viscosissima*

**Fig. 2.**
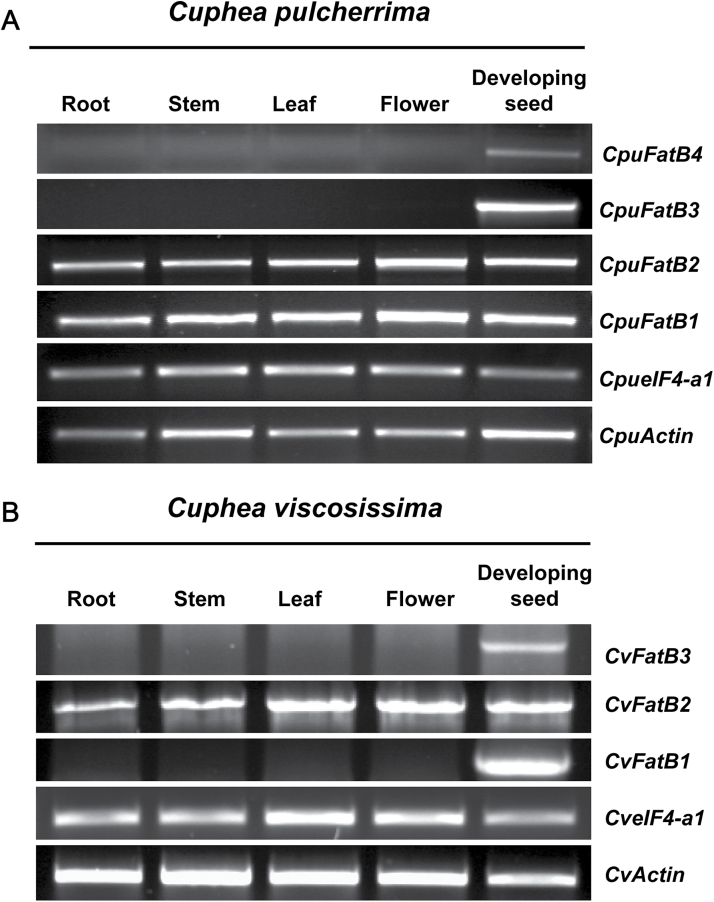
Spatial expression of FatB acyl-ACP thioesterases in *C. pulcherrima* and *C. viscosissima.* Total RNA was isolated from individual tissues and converted into cDNAs for RT-PCR analyses for evaluation of *FatB* gene expression in different tissues of *C. pulcherrima* and *C.viscossisima*. *Cuphea eIF4-a1* and actin genes were used as an internal control for RT-PCR.

Real-time PCR (RT-PCR) analysis was carried out with total RNAs isolated from roots, stems, leaves, flowers, and developing seeds of *C. pulcherrima* and *C. viscosissima* to examine relative expression levels of *FatB* transcripts from the 454 transcriptomic analyses. Among the four *FatB* genes from *C. pulcherrima*, *CpuFatB1* and *CpuFatB2* displayed ubiquitous expression in the tested organs, whereas *CpuFatB4* was expressed predominantly in developing seeds, and *CpuFatB3* was expressed exclusively in developing seeds (Fig. 2A). Among the three *FatB* genes in *C. viscosissima*, expression of *CvFatB1* and *CvFatB3* was detected only in developing seeds, while expression of *CvFatB2* was detected in all tested organs (Fig. 2B).

### Amino acid sequence comparisons are consistent with structure–function relationships related to substrate specificity among the *Cuphea* FatBs

**Fig. 3.**
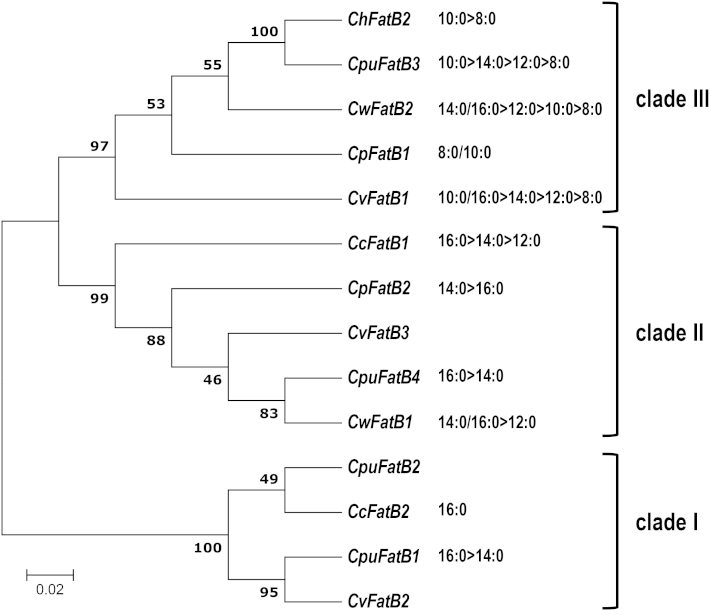
Phylogenetic tree of *Cuphea* FatB amino acid sequence clusters. Amino acid sequences of *Cuphea* FatBs were obtained from the protein database of the National Center for Biotechnology Information (NCBI). A phylogenic tree was built with the MEGA6 software, using the minimum-evolution method with 1000 bootstrap replications. *Cuphea calophylla* (CcFatB1, ABB71580; CcFatB2, ABB71581), *Cuphea wrightii* (CwFatB1, AAC49783; CwFatB2, AAC49784), *Cuphea viscosissima* (CvFatB1, AEM72522; CvFatB2, AEM72523; CvFatB3, AEM72524), *Cuphea palustris* (CpFatB1, 588563; CpFatB2, 1588564), *Cuphea hookeriana* (ChFatB2, AAC49269). Based on the literature and results presented herein, the relative substrate preferences of FatBs are indicated (e.g. 10:0 >8:0).

**Fig. 4.**
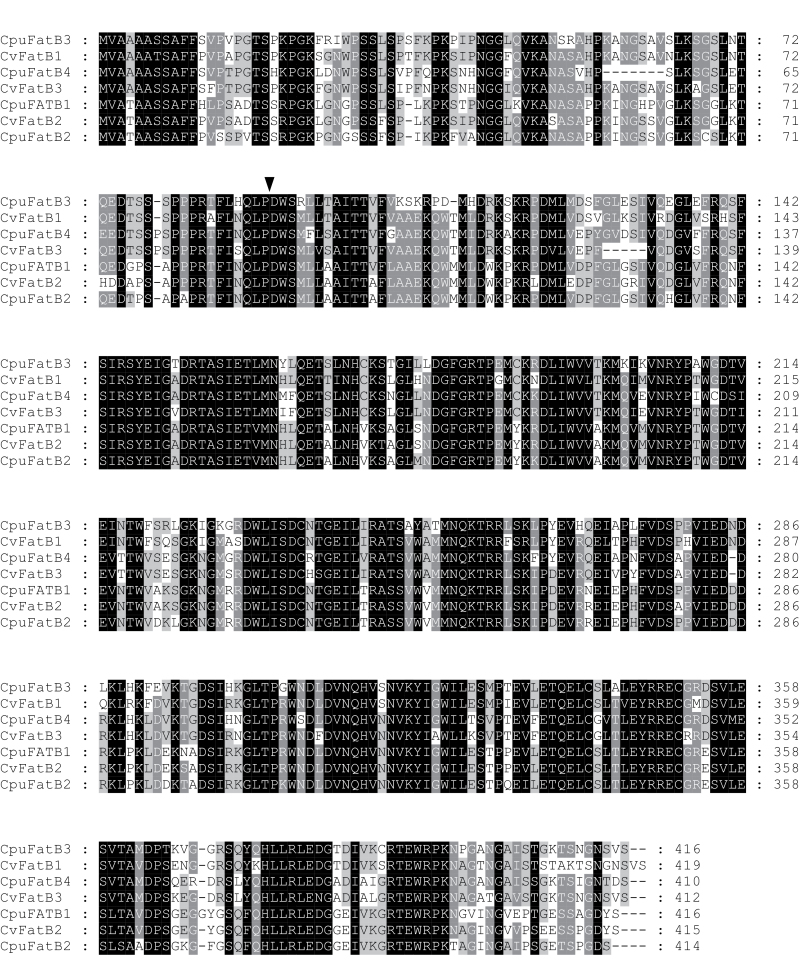
Alignment of deduced *Cuphea* FatB acyl-ACP thioesterases. Identical amino acids are shaded in black; conserved residues are shaded in grey. The triangle indicates the predicted site of the transit peptide.

Based on sequence homology and substrate chain length specificities, Cuphea FatB proteins have been classified into three groups: clade I generally has preferential activity towards 16:0-ACP; clade II generally has preferential activity towards 12:0-ACP and 14:0-ACP or broad-range specificity (12:0-ACP to 16:0-ACP); and clade III generally has preferential activity towards 8:0-ACP and 10:0-ACP (Voelker, 1996; Filichkin *et al.*, 2006; Jing *et al.*, 2011). Alignment of deduced FatBs from six *Cuphea* species showed that *C. pulcherrima* and *C. viscosissima FatB* genes could be grouped as seed-specific CpuFatB3 and CvFatB1 (clade III), CpuFatB4 and CvFatB3 (Clade II), and CpuFatB1, CpuFatB2, and CvFatB2 (clade I) (Fig. 3). The identity of amino acid sequences among *Cuphea* FatB polypeptides ranged from 70% to 93%. The chloroplast transit peptides were predicted based on homology with known FatBs (Fig. 4).

### Expression of *Cuphea FatB* genes in *Camelina* seed altered fatty acid profiles

Based on seed-specific expression in *Cuphea* and their phylogeny, *CpuFatB3* and *CpuFatB4* from *C. pulcherrima*, and *CvFatB1* and *CvFatB3* from *C. viscosissima* were chosen for evaluation to determine the ability to confer Jet A fuel-type fatty acid (C8–C16) production upon transgenic expression in *Camelina*. Previously reported medium chain-specific FatBs, *CpFatB2* from *C. palustris* (Dehesh *et al.*, 1996a), *UcFatB1* from California bay (Pollard *et al.*, 1991; Voelker *et al.*, 1996), and *ChFatB2* from *C. hookeriana* (Dehesh *et al.*, 1996b), were also tested (Table 1). *CpuFatB2* and *CvFatB2* were not chosen for *Camelina* transformation, due to their ubiquitous expression and classification as ‘typical’ FatBs with activity for 16:0-ACP. The cDNAs of eight *FatB* genes (*CpFatB2*, *UcFatB1*, *ChFatB2*, *CpuFatB1*, *CpuFatB3*, *CpuFatB4*, *CvFatB1*, and *CvFatB3*) were introduced into *Camelina* with strong seed-specific expression mediated by the soybean glycinin-1 promoter (Sims and Goldberg, 1989). Transgenic seeds were selected by fluorescence from the DsRed marker. Previously, transgenic rapeseed expressing *UcFatB1* showed a positive correlation between transgene copy number and lauric acid accumulation (Voelker *et al.*, 1996). However, multiple copy numbers of inserted transgenes have complex segregation patterns and might need many generations to establish homozygosity of a line (Mieog *et al.*, 2013). Also, multiple copies of the introduced gene have been implicated in transgene silencing (Tang *et al.*, 2007). To avoid these potential problems, 20–30 independent T_1_ lines were generated for each *FatB* gene, and then, based on the Mendelian segregation ratio of 3:1 (red seed:brown seed) of T_2_, single-copy insertion T_2_ lines with the highest content of MCFA in their seeds were selected for advancement to the T_3_ next generation for each FatB tested in transgenic *Camelina*.

**Table 1.** *Sources of FatB acyl-ACP thioesterase and LPAT for MCFA* in Camelina Cp, *Cuphea palustris*; Uc, *Umbellularia californica*; Ch, *Cuphea hookeriana*; Cpu, *Cuphea pulcherrima*; Cv, *Cuphea viscosissima*; NR, not reported.

Nomenclature	GenBank accession no.	Species	Fatty acid product of heterologous expression	Tissue specificity in source plant	Reported transgenic plants
*CpFatB2*	AAC49180	*C. palustris*	14:0 (*E. coli* and plant)	seed	*Arabidopsis thaliana* (Tjellström *et al.*, 2013)
*UcFatB1*	Q41635	*U. californica*	12:0 (*E. coli* and plant)	seed	*Brassica napus* (Voelker *et al.*, 1996; jellström *et al.*, 2013)
*ChFatB2*	AAC49269	*C. hookeriana*	8:0, 10:0 (*E. coli* and plant)	seed	*Brassica napus* (Dehesh *et al.*, 1996b; Tjellström *et al.*, 2013)
*CpuFatB1*	AGG79283	*C. pulcherrima*	16:0 (plant)	NR	*Camelina sativa* (Horn *et al.*, 2013)
*CpuFatB3*	AGG79285	*C. pulcherrima*	8:0, 10:0 (plant)	NR	*Arabidopsis thaliana* (Tjellström *et al.*, 2013)
*CpuFatB4*	AGG79286	*C. pulcherrima*	NR	NR	NR
*CvFatB1*	AEM72522	*C. viscosissima*	8:0, C10:0 (*E. coli*)	NR	NR
*CvFatB2*	AEM72523	*C. viscosissima*	14:0, C16:0, 16:1 (*E. coli*)	NR	NR
*CvFatB3*	AEM72524	*C. viscosissima*	14:0 (*E. coli*)	NR	NR
*CnLPAT*	XP002313814	*Cocos nucifera*	12:0 (Plant)	seed	*Brassica napus* (Knutzon *et al.*, 1999).

Similar to previous studies in *A. thaliana* and *B. napus* (Dehesh *et al.*, 1996a, b; Eccleston *et al.*, 1996; Tjellström *et al.*, 2013), *Camelina* seeds expressing *CpFatB2*, *UcFatB1*, and *ChFatB2* showed substrate specificities toward 14:0 (24 mol%), 12:0 (18 mol%), and 10:0 (10 mol%), respectively (Table 2). Although CpuFatB4 is most similar to clade II thioesterase which are broadly active on substrates ranging from 12:0-ACP to 16:0-ACP, the seeds of *CpuFatB4* transgenic lines accumulated 16:0 to 43.5 mol% of total fatty acid, a nearly 5-fold increase compared with non-transformed plants, and accumulated 14:0 to 8 mol% (Table 2). Expression of *CpuFatB3* resulted in the accumulation of 10:0 to 1.2 mol% of the total fatty acids as well as low levels of accumulation of 8:0, 12:0, and 14:0 in the engineered *Camelina* seeds. *CvFatB1*-expressing *Camelina* seeds had high levels of accumulation of 10:0 (9 mol%) and 16:0 (16 mol%), and low levels of 8:0, 12:0, and 14:0. Although *CpuFatB3* and *CvFatB1* are classified in clade III, they exhibit broad substrate specificity with ability to hydrolyse 10:0, 12:0, 14:0, and 16:0 acyl-ACPs. In contrast to its reported properties in *Escherichia coli* (Jing *et al.*, 2011), *CvFatB3* expression in *Camelina* seeds did not produce any detectable MCFAs, and fatty acid profiles resembled those of the wild type. This might be caused by nucleotide sequence differences at positions 442 and 1166 of the *CvFatB3* cDNA as described above (Table 2).

**Table 2.** *Fatty acid compositions (mol%) of seed lipids in transgenic* Camelina *expressing MCFA-specific FatB and in the wild type* The MCFA column is the total amount (mol%) of C8–C14 fatty acids, and the Jet FAs column is the total amount (mol%) of C8–C16 fatty acids with chain lengths found in Jet A and Jet A-1 fuels.

Genotype	8:0	10:0	12:0	14:0	16:0	18:0	18:1	18:2	18:3	20:0	20:1	22:1	MCFAs (C8–C14)	Jet FAs (C8–C16)
Wild type	–	–	–	–	8.9±0.9	4.0±0.7	10.6±0.7	25.1±1.1	40.5±2.0	1.6±0.0	7.5±0.5	1.8±0.1	0	8.9
CpuFatB1 (T_6_)	–	–	–	1.6±0.1	43.5±1.0	5.6±0.2	6.5±0.8	24.2±0.5	11.4±0.9	3.0±0.1	3.3±0.3	1.0±0.0	1.6	45.1
CpuFatB3 (T_2_)	0.5±0.1	1.2±0.2	0.2±0.0	1.0±0.1	8.3±0.3	4.6±0.6	21.8±2.3	18.0±1.2	27.1±2.6	2.7±0.5	12.0±0.8	2.5±0.3	2.9	11.2
CpuFatB4 (T_3_)	–	–	–	7.5±0.9	42.7±0.8	4.8±0.3	7.5±0.3	20.0±0.9	11.6±1.0	2.6±0.3	2.3±0.2	0.8±0.1	7.5	50.3
CvFatB1 (T_6_)	0.4±0.0	8.7±0.6	2.4±0.1	3.2±0.1	16.2±0.4	3.8±0.1	12.2±0.1	17.7±0.1	23.1±0.7	2.9±0.1	7.0±0.4	2.4±0.1	14.7	30.9
CvFatB3 (T_2_)	–	–	–	–	9.3±0.7	3.9±0.4	13.3±0.2	22.8±1.5	33.1±2.0	2.5±0.2	11.8±1.1	3.3±0.3	0	9.3
CpFatB2 (T_7_)	–	–	–	23.8±0.8	18.1±0.9	3.3±0.4	5.6±0.9	15.5±0.9	26.8±3.1	1.4±0.2	4.2±0.4	1.3±0.2	23.8	41.9
UcFatB1 (T_7_)	–	–	18.4±0.9	2.7±0.1	7.1±0.2	4.0±0.3	8.1±0.7	29.9±0.6	21.5±1.3	1.8±0.2	5.1±0.3	1.2±0.1	21.1	28.2
ChFatB2 (T_7_)	0.7±0.1	10.3±0.4	0.9±0.0	0.7±0.0	9.0±0.3	4.7±0.3	14.8±0.6	30.0±0.3	19.9±0.8	1.9±0.1	5.8±0.1	1.2±0.0	12.6	21.6
CpFatB2+UcFatB1 (T_4_)	–	–	9.4±0.8	16.0±0.3	11.2±0.4	2.2±0.1	6.2±0.1	23.7±1.0	22.1±0.7	1.8±0.1	5.7±0.1	1.7±0.0	25.4	36.5
CpFatB2+ChFatB2 (T_4_)	–	8.7±0.5	0.8±0.5	9.3±0.0	12.4±0.2	3.4±0.0	21.6±0.9	16.2±0.4	16.1±0.5	2.1±0.0	7.4±0.1	1.9±0.0	18.8	31.1
CpFatB2+UcFatB1+ChFatB2 (T_4_)	–	8.5±0.9	1.6±0.5	9.6±0.2	12.0±0.2	3.3±0.1	21.4±0.3	16.9±0.7	15.8±0.2	1.9±0.0	7.1±0.2	1.8±0.1	19.7	31.8
CpFatB2+CnLPAAT (T_4_)	–	–	–	36.9±1.9	16.8±0.6	3.0±0.3	3.1±0.3	19.2±0.6	16.0±2.0	1.9±0.2	2.3±0.2	0.9±0.1	36.9	53.7
UcFatB1+CnLPAAT (T_4_)	–	–	28.4±1.5	3.8±0.2	5.6±0.4	3.1±0.6	7.4±0.4	13.6±0.3	30.4±0.7	1.2±0.1	4.9±0.3	1.5±0.1	32.2	37.8
ChFatB2+CnLPAAT (T_4_)	0.5±0.1	9.7±0.6	0.8±0.0	0.7±0.0	9.3±0.2	5.0±0.4	13.1±0.4	31.3±0.6	20.0±0.6	2.2±0.1	6.0±0.5	1.4±0.2	11.7	21.0

Values are the means±SD of five biological replicates.

The number in parentheses (T_x_) indicates the generation of the seeds.

Two and three *FatB* cDNAs were co-expressed to produce more diverse MCFA compositions in *Camelina* seeds to mimic more closely the carbon chain lengths of Jet A fuel (from 8- to 16-carbon distribution) and to examine the utility of this approach for enhancing MCFA accumulation in *Camelina* seeds relative to expression of individual FatBs. Co-expression of *CpFatB2* and *UcFatB1* resulted in the production of 12:0, 14:0, and 16:0 (Table 2). Co-expression of *CpFatB2* with *ChFatB2* or three cDNAs (*CpFatB2*, *UcFatB1*, and *ChFatB2*) produced fatty acids ranging from 8:0 to 16:0. Although diverse compositions of MCFAs were observed in seeds of the co-expression lines, the amounts of each MCFA were less than those obtained with expression of individual cDNAs (Table 2). For example, 12:0 made up 18 mol% of the total fatty acids in seeds of *UcFatB1*-expressng lines, and the amount of 14:0 in seeds of *CpFatB2*-expressing lines was 24 mol% of total fatty acids (Table 2). In contrast, seeds of *CpFatB2* and *UcFatB1* co-expression lines accumulated only 7 mol% of 12:0 and 12 mol% of 14:0 (Table 2). Myristic acid (14:0) content was also reduced in seeds of *CpFatB2* and *ChFatB2* co-expression lines and *CpFatB2*, *UcFatB1*, and *ChFatB2* co-expression lines by nearly 3-fold relative to seeds expressing only *CpFatB2*. Similarly, lauric acid (12:0) content was reduced by ~9-fold in seeds of lines co-expressing *CpFatB2*, *UcFatB1*, and *ChFatB2* compared with seeds expressing *UcFatB1* alone (Table 2).

**Fig. 5.**
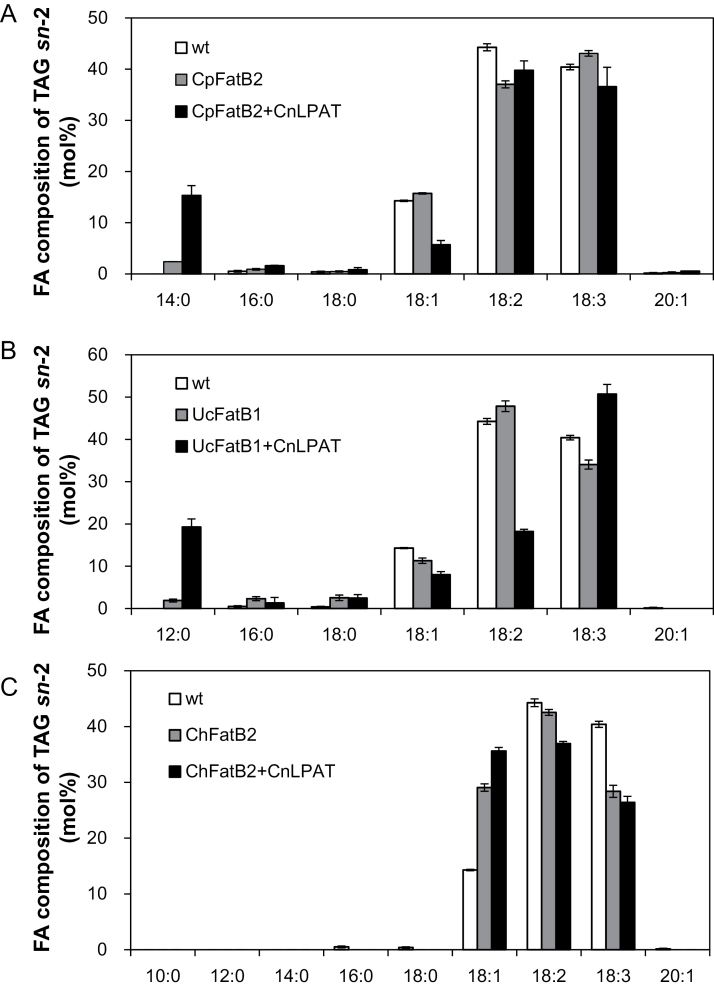
Fatty acid composition of the *sn*-2 position of seed oil TAG. Fatty acid composition of the *sn*-2 position of seed oil TAG in plants expressing *FatB* genes alone or in combination with *CnLPAT* as determined by lipase digestion-based analyses. The data represent averages of four biological replicates ±SD. (A) *CpFatB2* and *CpFatB2* with *CnLPAT*. (B) *UcFatB1* and *UcFatB1* with *CnLPAT*. (C) *ChFatB2* and *ChFatB2* with *CnLPAT*.

To increase MCFA levels further in *Camelina* seeds, FatB polypeptides were co-expressed with a coconut lysophosphatidic acid acyltransferase (CnLPAT) that has been shown to insert 12:0 at the *sn*-2 position of the TAG glycerol backbone (Table 1), leading to 12:0 accumulation in transgenic *B. napus* seeds (Knutzon *et al.*, 1999). Co-expression of either *CpFatB2* or *UcFatB1* with *CnLPAT* in *Camelina* resulted in further accumulation of MCFAs in the seed (Table 2). However, co-expression of *CnLPAT* with the C8/C10-specific FatB *ChFatB2* did not increase 8:0 or 10:0 fatty acid levels in *Camelina* seeds relative to expression of *ChFatB2* alone (Table 2). Analysis of *sn*-2 fatty acids of TAG confirmed the effect of *CnLPAT* to increase MCFAs in TAG. Myristic acid (14:0) and lauric acid (12:0) were detected in the *sn*-2 position of TAG in the co-expression lines of *CpFatB2* with *CnLPAT* and *UcFatB1* with *CnLPAT*, respectively, while these fatty acids were 5- to 10-fold lower in the absence of *CnLPAT* (Fig. 5A, B). Interestingly, 10:0 was not detected at the *sn*-2 position of TAG in *Camelina* seeds co-expressing *ChFatB2* and *CnLPAT* (Fig. 5C), indicating that CnLPAT has substrate specificity for CoA esters of 12:0 preferentially and 14:0 to a lesser extent, but is not active with 10:0-CoA (Table 2).

**Fig. 6.**
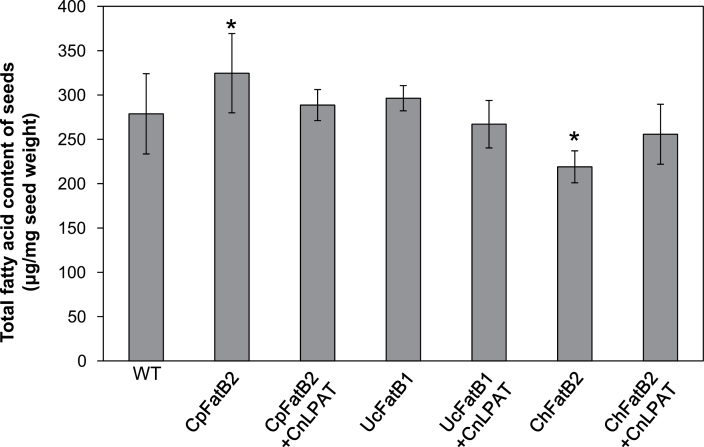
Total fatty acid contents of engineered *Camelina* lines. Total fatty acid contents of seeds from FatB with or without C*nLPAT* expression were analysed by gas chromatography. The data represents means ±SD with five biological replicates. Asterisks indicate statistical differences compared with the wild type (*P<0.05).

Similar to a previous report by Tjellström *et al.* (2013), a positive correlation between 8:0 and 10:0 levels and 18:1 levels was observed. Seeds expressing *CpuFatB3*, for example, had a 2-fold increase of 18:1. However, although 12:0, 14:0, and 16:0 increase in seeds expressing *UcFatB1*, *CpFatB2*, and *CpuFatB1*, the level of 18:1 decreased to 8, 6, and 7 mol%, respectively, compared with wild-type seeds that had 11 mol% of 18:1 (Table 2). Furthermore, total fatty acid content in 14:0-accumulating seeds expressing *CpFatB2* was increased, whereas a decrease in total fatty acids was detected in seeds of 10:0-producing FatBs, in comparison with wild-type seeds (Supplementary Fig. S1 at *JXB* online). Notably, co-expression of MCFA-specific *FatB* and *CnLPAT* resulted in an increase of MCFA without significant impact on total seed fatty acid content under greenhouse growth conditions (Fig. 6). Interestingly, lines expressing the C10-producing *ChFatB2* thioesterase had lower seed fatty acid content than wild-type controls, but this was restored to wild-type levels with co-expression of *CnLPAT* (Fig. 6).

### Disruption of *KASII* in *Camelina* seeds increases palmitic acid content but decreases MCFA content in FatB expression lines

Seed-specific suppression of *Camelina KASII* was examined as an additional strategy to increase palmitic acid (16:0) and MCFA content to mimic Jet A fuel composition. *KASII* encodes β-ketoacyl-ACP synthase that catalyses the initial step in the elongation of 16:0-ACP to 18:0-ACP, and *KASII* suppression has been shown to enhance the 16:0 content of seeds (Pidkowich *et al.*, 2007). Five seed-specific *KASII*-RNAi lines (Supplementary Fig. S1 at *JXB* online) were chosen for detailed characterization based on the increased 16:0 content of their seed oil and normal phenotype. Seeds from homozygous T_4_ lines had 26–28 mol% 16:0, nearly 3-fold higher compared with non-transformed lines (Table 3). *KASII*-RNAi transgenic *Camelina* seeds showed no significant changes in total seed oil content relative to the wild type (Table 3). Reduced transcript levels of *CsKASII* in developing seeds of *CsKASII*-RNAi lines were not detected in comparison with the wild type (Supplementary Fig. S3). In this regard, complete *KASII* knock-out is lethal and high levels of palmitic acid (16:0) in KASII-RNAi lines lead to aborted ovules in *Arabidopsis* (Pidkowich *et al.*, 2007). In the present study, transgenic *KASII*-RNAi lines with increased 16:0 but normal growth phenotypes were selected. Because increased 16:0 and 16:1 is an obvious phenotype of *KASII*-RNAi, the altered TAG composition of *CsKASII*-RNAi provides evidence for the partial suppression of CsKASII activity.

**Table 3.** *Fatty acid composition (mol%) of seed lipids in wild-type* Camelina *and transgenic* Camelina *of* CsKASII*-RNAi* Values are the means ±SD of five biological replicates.

Genotype	16:0	16:1	18:0	18:1	18:2	18:3	20:0	20:1	22:1	Total fatty acid content (μg mg^–1^ seed weight)
Wild type	10.1±0.4	0.2±0.0	4.4±0.2	14.2±0.4	34.0±0.3	20.7±1.0	2.8±0.1	10.7±0.3	2.7±0.1	222.4±11.2
CsKASII-RNAi #1–3 (T_3_)	27.9±0.5	4.1±0.0	3.7±0.2	6.7±0.5	19.2±1.2	23.6±1.1	3.6±0.1	8.3±0.1	2.8±0.1	243.4±4.7
CsKASII- RNAi #2–6 (T_3_)	27.9±0.8	4.1±0.2	3.7±0.3	6.5±0.1	17.4±1.6	25.8±0.9	3.4±0.2	8.5±0.2	2.0±0.1	263.0±21.0
CsKASII- RNAi #5–2 (T_3_)	26.3±0.2	3.5±0.1	3.8±0.1	7.1±0.2	20.4±0.9	23.8±0.9	3.6±0.0	8.7±0.2	2.7±0.1	247.6±3.4
CsKASII- RNAi #6–1(T_3_)	28.2±0.2	4.3±0.1	3.7±0.2	6.3±0.1	17.0±0.2	25.4±0.4	3.6±0.2	8.4±0.1	2.9±0.1	263.6±10.4

T_3_ refers to the seed generation analysed.

**Fig. 7.**
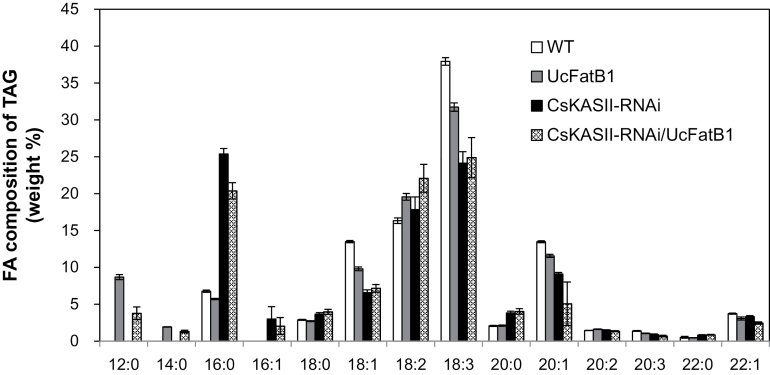
Fatty acid composition (weight%) of seed lipids in *CsKASII*-RNAi *Camelina* expressing *UcFatB1*. Fatty acids were extracted from transgenic *Camelina* seeds and analysed using gas chromatography. Values are the means ±SD of five biological replicates.

To determine whether *KASII* suppression increases MCFA levels, the *KASII*-RNAi construct was transformed into a *Camelina* line homozygous for the *UcFatB1* transgene. The resulting 12:0 content in seeds from these lines ranged from 3 mol% to 7 mol% versus 15 mol% (or 5% versus 10% on a weight percent basis to total oil) in the parent line lacking *KASII*-RNAi suppression (Table 4; Fig. 7). The mol% of C8–C16 fatty acids in *KASII*-RNAi lines was not significantly affected by suppression in either the wild type or the UcFatB1 background (Table 4), and ranged from 24.5% to 33.7% of total fatty acid in both backgrounds. These results suggested that efficient downstream acylation of MCFAs into TAG is important for the stable accumulation of MCFAs in the transgenic *Camelina* seeds. In this regard, with high levels of 16:0 production, it is likely that 16:0-CoA is used in preference to 12:0-CoA for acylation on the glycerol backbone.

**Table 4.** *Fatty acid composition (mol%) of seed lipids in* CsKASII*-RNAi* Camelina *expressing* UcFatB1 Values are the means ±SD of five biological replicates. The Jet FAs column is the total amount (mol%) of C8–C16 fatty acids with chain lengths found in Jet A and Jet A-1 fuels.

Genotype	12:0	14:0	16:0	16:1	18:0	18:1	18:2	18:3	20:0	20:1	22:1	Jet FAs (C8–C16)
UcFatB1(T_7_)	15.1±2.0	2.7±0.5	6.7±0.5	0.0±0.0	4.2±0.5	10.7±1.5	26.7±1.9	19.3±2.6	3.5±0.2	8.7±0.5	2.5±0.1	24.5
CsKASII-RNAi (T_4_)	0.0±0.0	0.0±0.0	28.3±0.9	3.4±1.9	3.7±0.2	6.7±0.4	18.3±1.8	24.9±1.6	3.5±0.2	8.5±0.2	2.8±0.1	28.3
CsKASII-RNAi/ UcFatB1 #1–2 (T_3_)	5.5±1.7	1.6±0.2	24.6±1.6	2.6±0.7	4.0±0.4	6.7±0.4	24.6±1.5	19.5±3.4	3.4±0.2	5.7±0.6	1.9±0.5	31.6
CsKASII-RNAi/ UcFatB1 #2-1 (T_3_)	5.3±1.2	1.6±0.2	20.2±1.2	2.8±1.3	3.7±0.3	6.8±0.5	21.2±1.9	24.8±2.8	3.9±0.3	7.3±2.7	2.5±0.1	27.1
CsKASII- RNAi/ UcFatB1 #8-1 (T_3_)	7.5±2.4	2.0±0.6	24.2±2.3	2.4±0.6	4.1±0.5	6.0±0.2	21.3±3.8	20.1±3.5	4.1±0.4	6.0±0.4	2.3±0.3	33.7
CsKASII- RNAi/ UcFatB1 #9-1 (T_3_)	3.0±1.1	1.3±0.1	22.4±2.2	1.9±0.5	3.6±1.1	7.1±0.5	25.0±3.0	22.7±2.4	3.5±0.5	7.1±1.9	2.4±0.5	26.7
CsKASII- RNAi/ UcFatB1 #10–1 (T_3_)	4.4±1.4	1.2±0.3	19.7±2.4	1.2±0.5	5.3±0.9	8.9±0.7	26.5±1.1	18.6±0.9	4.1±0.4	7.9±1.0	2.2±0.4	25.3
CsKASII- RNAi/ UcFatB1 #12-1 (T_3_)	4.9±1.2	1.5±0.3	19.9±0.1	1.3±0.1	4.8±0.8	8.3±0.8	28.0±2.5	19.2±2.3	4.2±0.6	5.7±1.3	2.0±0.3	26.4
CsKASII- RNAi/ UcFatB1 #15-1 (T_3_)	3.9±0.7	1.2±0.3	21.8±1.9	1.6±0.6	4.4±0.7	8.7±2.3	27.8±3.4	18.3±3.9	3.7±0.3	6.5±0.4	2.0±0.6	26.9

T_7_ and T_3_ refer to the seed generation analysed.

### Acyl-ACP pool analysis of developing *Cuphea* and *Camelina* seeds

**Fig. 8.**
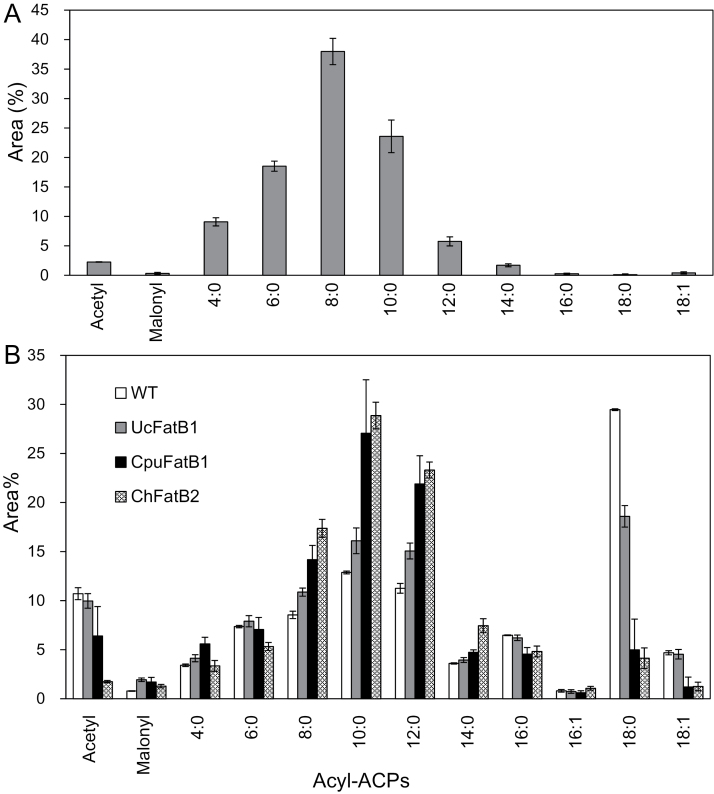
Acyl-ACP analysis of developing seeds of *Cuphea viscosissima* and wild-type and transgenic *Camelina* seeds. Shown in (A) are acyl-ACP pools in developing seeds of *C. viscosissima*. Shown in (B) are acyl-ACP profiles in wild-type *Camelina* (WT) and transgenic *Camelina* seeds expressing UcFATB1, CpuFATB1, and ChFatB2 at 15 DAF. The data are means ±SD of five biological replicates.

The expression of *FatB* genes from *Cuphea* results in increased MCFA levels in *Camelina* seed oils, but does not approach the levels of MCFA that occur naturally in *Cuphea* seeds. This may be due to the evolution of substrate specificity within the components of the FAS complex in *Cuphea* species. To examine this hypothesis, the acyl-ACP composition of developing seeds from *C. viscosissima* and *Camelina* was analysed by electrospray ionization-tandem mass spectrometry (ESI-MS/MS) (Fig. 8). Acyl-ACP pool sizes were estimated based on comparison of relative peak area percentages per unit of protein analysed. In *C. viscosissima*, 8:0-ACP made up 40% of the acyl-ACP pools, followed by 10:0-ACP (24%) and 6:0-ACP (19%). Long-chain (≥16) acyl-ACPs were barely detected and made up <1% of total acyl-ACP in developing seeds of *C. viscosissima*. In contrast, acyl-ACP pools of transgenic and wild-type *Camelina* seeds were increased at 10 days after flowering (DAF) (Supplementary Fig. S4 at *JXB* online). However, increased percentages of 8:0, 10:0, and 12:0 ACPs were detected in 15 DAF developing seeds from FatB transgenic lines compared with wild-type seeds.

## Discussion

The goal of this study was to generate MCFA-rich seed oils for use as feedstocks for jet fuel applications. To identify *FatB* cDNAs capable of generating novel MCFA-rich oil compositions, 454 transcriptomic studies of developing seeds from two *Cuphea* species was performed. To engineer oilseed with altered fatty acid composition, candidate *FatB* cDNAs were expressed individually or in combination with an MCFA-CoA-specific LPAT in *Camelina*. To increase levels of MCFA further, down-regulation of *KASII* in combination with overexpression of MCFA-specific *FatB* genes was also performed. From this research, two *FatB* cDNAs, *CpuFatB3* and *CvFatB1*, were identified from transcriptomics data that were capable of generating 10:0 in *Camelina* seeds. Also identified were two FatBs (*CpuFatB2* and *CvFatB2*) ubiquitously expressed in *Cuphea*, as well as a seed-specific FatB (*CpuFatB4*) that produced oils enriched in 16:0 and lesser amounts of 14:0 upon expression in *Camelina* seeds. In addition, co-expression of *FatB* cDNAs with different substrate specificities generated oils with a range of MCFAs, but the accumulation of each MCFA was less than that obtained by the expression of individual FatBs. Furthermore, co-expression of the MCFA-CoA specific coconut LPAT increased overall levels of 12:0 and 14:0 accumulation in *Camelina* seeds and the amounts of these fatty acids in the *sn*-2 position of *Camelina* seed TAGs. This approach did not increase 10:0 at the *sn*-2 position of *Camelina* seed TAGs, suggesting that the coconut LPAT uses 10:0-CoA poorly as a substrate. While suppression of *KASII* was effective in blocking elongation of 16:0 in *Camelina* seeds, 12:0 accumulation in *Camelina* seeds from expression of a 12:0-ACP-specific FatB was not enhanced by this approach.

Although *C. pulcherrima* accumulates almost exclusively 8:0 fatty acids in its seed oil, none of the *FatB* cDNAs isolated from developing seeds of this plant was capable of generating >1 mol% 8:0 upon expression in developing *Camelina* seeds. Seed-specific expression of *CpuFatB4* in *Camelina* seeds, for example, yielded primarily 16:0 and lower amounts of 14:0, and seed-specific *CpuFatB3* expression in *Camelina* seeds produced low levels of 10:0 and lower amounts of 8:0, 12:0, and 14:0. Similar results were previously observed with *CcFatB1* from *C. calophylla* seeds, which accumulate ~60% 12:0 (Filichkin *et al.*, 2006). Expression of this thioesterase in *Arabidopsis* seeds resulted in the accumulation of 16:0 to ~20 mol% of the total fatty acids, but yielded only ~1.5 mol% of 12:0. However, co-expression of *CcFatB1* with a *Cuphea wrightii* β-keto-acyl-ACP synthase in *Arabidopsis* seeds shifted fatty acid accumulation to ~13 mol% of 12:0 with only a small elevation in 16:0 content compared with non-transformed plants. These results, along with the present results for *C. pulcherrima* and *C. viscosissima* FatBs, indicate that the high content of specific MCFAs in *Cuphea* seeds is determined not only by FatBs but also by their acyl-ACP substrate pools in these seeds resulting from specialization in FAS. Comparative measurements of acyl-ACP pools in seeds of *C. viscosissima* developing seeds and those of wild-type and transgenic *Camelina* seeds engineered for *Cuphea* FatB expression support this hypothesis (Fig. 8). The most notable difference between developing *Cuphea* and *Camelina* seeds was the near absence of ≥C16 acyl-ACPs in *C. viscosissima* seed and an enrichment of 8:0- and 10:0-ACP in these seeds, in part consistent with the high levels of 8:0 and 10:0 accumulation in *C. viscosissima* seeds. Conversely, long-chain acyl-ACPs were more abundant in developing wild-type *Camelina* seeds. The reduction of ≥C16 acyl-ACPs in developing *Camelina* seeds expressing CpuFatB1 and ChFatB2 relative to developing wild-type *Camelina* seeds (Fig. 8) may be indicative of an efficient termination of acyl-ACP chain elongation by the activities of these enzymes.

In addition to potential bottlenecks in FAS that limit the production of 8:0 and 10:0 fatty acids in divergent FatB-expressing *Camelina* seeds, the present results, as well as those of others, show that downstream acylation of MCFAs on the TAG glycerol backbone limits accumulation of these fatty acids in the transgenic *Camelina* seeds. Unlike other unusual fatty acids, the accumulation of MCFAs is believed to follow the traditional Kennedy pathway, which involves the sequential acylation of the *sn*-1 of glycerol-3-phosphate (G3P) and the *sn*-2 of lysophosphatidic acid (LPA) with acyl-CoA substrates to produce phosphatidic acid (PA) by GPAT and LPAT, respectively. Then PA is converted to diacylglycerol (DAG), and finally the *sn*-3 of DAG is acylated to form TAG. The importance of specialized acyltransferases for MCFA accumulation was most evident with the co-expression of *UcFatB1* with *CnLPAT*, which resulted in a nearly 10 mol% increase in 12:0 accumulation compared with expression of *UcFatB1* alone. As expected for a specialized LPAT, this increased accumulation was largely due to enhanced amounts of 12:0 at the *sn*-2 position of the TAG glycerol backbone (Fig. 5). The coconut CnLPAT, however, showed little ability to introduce 10:0 formed by ChFatB2 into the *sn*-2 position of the TAG glycerol backbone (Fig. 5C), similar to results previously described in transgenic *B. napus* (Dehesh, 2001). Given the 8:0- and 10:0-rich fatty acid profiles of *C. pulcherrima* and *C. viscosissima* seeds, LPATs and other acyltransferases, particularly DGATs (Bafor and Stymne, 1992; Wiberg *et al.*, 1994, 2000), with activity for 8:0 and 10:0 MCFA substrates are expected to occur. Characterization of candidate acyltransferases from *C. pulcherrima* and *C. viscosissima* seed transcriptomes is currently a major focus of research.

Consistent with previously reported studies in *Arabidopsis* (Tjellström *et al.*, 2013), some transgenic *Camelina* lines producing 8:0 and 10:0 (e.g. CpuFatB3 and CvFatB1) showed increased levels of 18:1 and decreased levels of 18:2, suggesting that the *Camelina* FAD2 is less active on 18:1 when paired with 8:0 or 10:0 on phosphatidylcholine (PC). However, other transgenic lines (e.g. ChFatB2) showed the opposite phenotype: an increase in 18:2. Therefore, the question of alternative pathways for trafficking MCFA into TAG remains unclear and merits further investigation.

Recently, plastid acyl-ACP synthetase, known as an acyl activating enzyme (AAE), has been proposed to be involved in recycling MCFAs into the plastid for further elongation (Tjellström *et al.*, 2013). Exogenous fatty acids have different metabolic fates depending on their chain length and level of unsaturation. For example, a shorter chain length (≤C10) was used preferentially in plastids for synthesis of plastidal lipid, whereas longer substrates (≥C14) were predominantly utilized in cytosolic lipids (Roughan *et al.*, 1987). This activation of MCFAs to ACP, not only added exogenously but also synthesized in plastids, occurs directly via a plastid acyl-ACP synthetase (Koo *et al.*, 2005; Tjellström *et al.*, 2013). The effect of the limiting acyl-ACP synthetase activity for the accumulation of MCFA was significant on 8:0 which was increased by almost 2-fold in *aae 15/16* seeds expressing *CpuFatB3* (Tjellström *et al.*, 2013). It is interesting to note that it was not possible to find homologues of *Arabidopsis* AAE15 (or AAE16) in the *Cuphea* transcriptome database used here. This suggests that the presence of acyl-ACP synthetases in *Camelina* seeds may be an additional bottleneck for MCFA accumulation, particularly 8:0 and 10:0 accumulation.

The main problems of present biodiesel include several technical aspects, such as cold flow properties and oxidative stability, as well as feedstock availability and cost (Knothe, 2014). Due to their unique fatty acid composition, *Cuphea* seed oils have received attention as a source of biodiesel (Geller *et al.*, 1999; Knothe *et al.*, 2009; Lovestead *et al.*, 2010; Knothe, 2014). The evaluation of fuel properties, such as the cetane number, kinematic viscosity, and oxidative stability, showed that biodiesel derived from *Cuphea* oil had properties superior to those of other vegetable oils for biodiesel fuels (Knothe *et al.*, 2009). In particular, plant oils rich in 10:0 would be more desirable for biodiesel production than conventional plant oil (Knothe *et al.*, 2009). In addition, *Cuphea* species represent a deep reservoir of divergent FatBs with a range of substrate specificities for generating oils that mimic the C8–C16 hydrocarbon component of Jet A and Jet A-1 fuels. As shown here, multiple FatBs with differing substrate specificities can be co-expressed to obtain *Camelina* seed oil with a broad mixture of fatty acid chain lengths ranging from C8 to C16. However, the expression of multiple FatBs was not as effective at generating amounts of individual MCFAs as obtained by expression of single FatBs. While this may be due to suboptimal acyl-ACP pool compositions to match the substrate preferences of divergent FatBs, it cannot be excluded that mixed FatBs might form heterodimers that are less active than homodimers. It is known that plant FatBs function as dimers (McKeon and Stumpf, 1982; Hellyer *et al.*, 1992). Based on structural similarity to other thioesterases, the active sites of the acyl-ACP thioesterase are predicted to lie at the dimer interface (Dillon and Bateman, 2004), and heterodimerization may affect the active site architecture to reduce activity

Overall, the present findings highlight the need for a more complete understanding of *Cuphea* FAS to tailor acyl-ACP pools predictably in host oilseeds to match the substrate properties of introduced FatBs and to maximize production of individual or mixtures of MCFAs for uses such as biodiesel and jet fuel production. In addition to *FatB* sequences, the transcriptomic analyses of developing *C. pulcherrima* and *C. viscosissima* seeds uncovered a wealth of FAS genes that will be useful for co-expression in *Camelina* to re-create a *Cuphea*-type FAS that possibly has higher 8:0- and 10:0-ACP substrate pools for divergent FatBs. As indicated by the present inability to increase Jet FA accumulation by suppression of the *Camelina KASII*, altering native FAS in the host oilseed is likely to be insufficient on its own for generating a specialized FAS for high levels of Jet FA production.

## Supplementary data

Supplementary data are available at *JXB* online.

Figure S1. Total fatty acid content of engineered *Camelina* lines.

Figure S2. 16:0 Fatty acid composition of *CsKASII*- RNAi TAG.

Figure S3. Transcript level of *CsKASII*-RNAi in developing seeds.

Figure S4. Acyl-ACP analysis.

Table S1. List of primers used in this study.

Supplementary Data

## Abbreviations:

ACP: acyl carrier protein
DsRed: *Discosoma* sp. red fluorescent protein
FAMEs: fatty acyl methyl esters
FAS: fatty acid synthase
FatB: FatB acyl-ACP thioesterase
GC: gas chromatography
KASII: β-ketoacyl-acyl carrier protein synthase II
LPAT: lysophosphatidic acid acyltransferase
MCFA: medium-chain fatty acid
TAG: triacylglycerol.
