# Supplementary material for: Toward production of jet fuel functionality in oilseeds: identification of FatB acyl-acyl carrier protein thioesterases and evaluation of combinatorial expression strategies in Camelina seeds
Source: J Exp Bot. 2015 May 11;66(14):4251–65. doi: 10.1093/jxb/erv225 (PMC4493788; doi:10.1093/jxb/erv225)
Supplement: Supplementary Data [file supp_66_14_4251__index.html]

Toward production of jet fuel functionality in oilseeds: identification of FatB acyl-acyl carrier protein thioesterases and evaluation of combinatorial expression strategies in Camelina seeds — Toward production of jet fuel functionality in oilseeds: identification of FatB acyl-acyl carrier protein thioesterases and evaluation of combinatorial expression strategies in Camelina seeds — Toward production of jet fuel functionality in oilseeds: identification of FatB acyl-acyl carrier protein thioesterases and evaluation of combinatorial expression strategies in Camelina seeds — Supplementary Data 

# Toward production of jet fuel functionality in oilseeds: identification of FatB acyl-acyl carrier protein thioesterases and evaluation of combinatorial expression strategies in *Camelina* seeds

## Supplementary Data

Data files

**Files in this Data Supplement:**

- Supplementary Data - Supplementary Data
