# Supplementary material for: Toward production of jet fuel functionality in oilseeds: identification of FatB acyl-acyl carrier protein thioesterases and evaluation of combinatorial expression strategies in Camelina seeds
Source: J Exp Bot. 2015 May 11;66(14):4251–65. doi: 10.1093/jxb/erv225 (PMC4493788; doi:10.1093/jxb/erv225)
Supplement: Supplementary Data [file supp_erv225_jexbot149344_file001.pdf]

Supplementary Table 1. List of primers used in studies.

Primer name	Primer sequence	Purpose
CpueIF4-RT-F	GGTGAAGCGTGACGAACTGAC	Internal control of *C. pulcherrima* and *C. viscosissima* for RT-PCR
CpueIF4-RT-R	CTCTAGTGTTCTGGTCCATGTCTCC	
CpuActin-F	GGGATGACATGGAGAAGATCTGG	Internal control of *C. pulcherrima* for RT-PCR
CpuActin-R	GGAATCTTTCAGCACCGATGGT	
CvActin-F	GGGATGACATGGAGAAGATCTGG	Internal control of *C. viscosissima* for RT-PCR
CvActin-R	GTTCATAGTCAAGGGCGACATAGG	
CpuFatB1-5'EcoRI	gactgaattcATGGTGGCTACCGCTGCAAGTT	Overexpression and RT-PCR
CpuFatB1-3'XbaI	gacttctagaCTAAGAGTAGTCTCCAGCCGAGG	
CpuFatB2-F	gactgaattcATGGTGGCTACCGCTGCAAGCT	RT-PCR
CpuFatB2-R	gactctcgagCTAAGAGTCTCCAGGTGAGGTCTCC	
CpuFatB3-5'EcoRI	gactgaattcATGGTGGCTGCTGCAGCAA	Overexpression and RT-PCR
CpuFatB3-3'XbaI	gacttctagaCTAAGAGACCGAGTTTCCATTTGAA	
CpuFatB4-5'EcoRI	gactgaattcATGGTGGCTGCCGCAGC	Overexpression and RT-PCR
CpuFatB4-3'XbaI	gacttctagaCTAAGAGTCAGTGTTTCCAATTGAAGTC	
CvFatB1-5'EcoRI	gactgaattcATGGTGGCTGCTGCAGC	Overexpression and RT-PCR
CvFatB1-3'XbaI	gacttctagaCTAAGAGACCGAGTTTCCATTTGAAG	
CvFatB2-F	ATGGTGGCTACCGCTGCAA	RT-PCR
CvFatB2-R	CTAAGAGTAGTCTCCAGGTGAGGAC	
CvFatB3-5'EcoRI	gactgaattcATGGTGGCTGCCGCAGC	Overexpression and RT-PCR
CvFatB3-3'XbaI	gacttctagaCTAAGAGACGGAATTTCCATTTGAA	

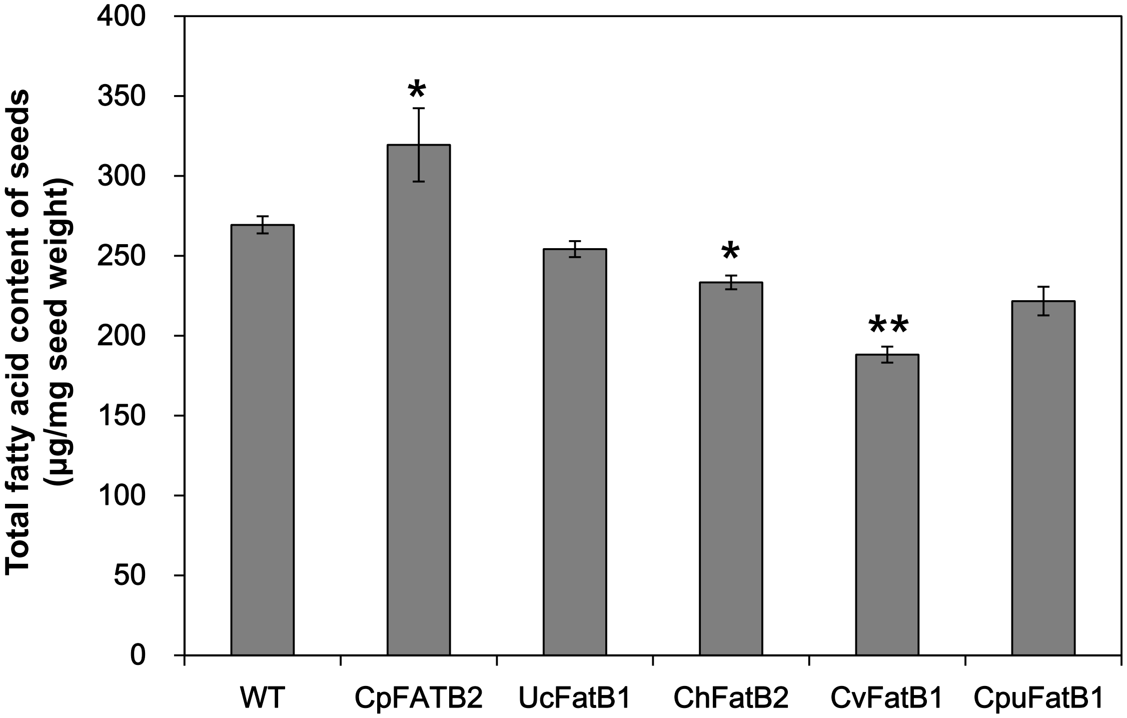

Figure S1. Total fatty acid content of engineered Camelina lines.
Total fatty acid content of seeds from FatB transgenic plants were analyzed by GC. The data represents means ± SD of five biological replicates. Airstrikes indicate statistical differences compared to wild type (*P<0.05, **P<0.01).

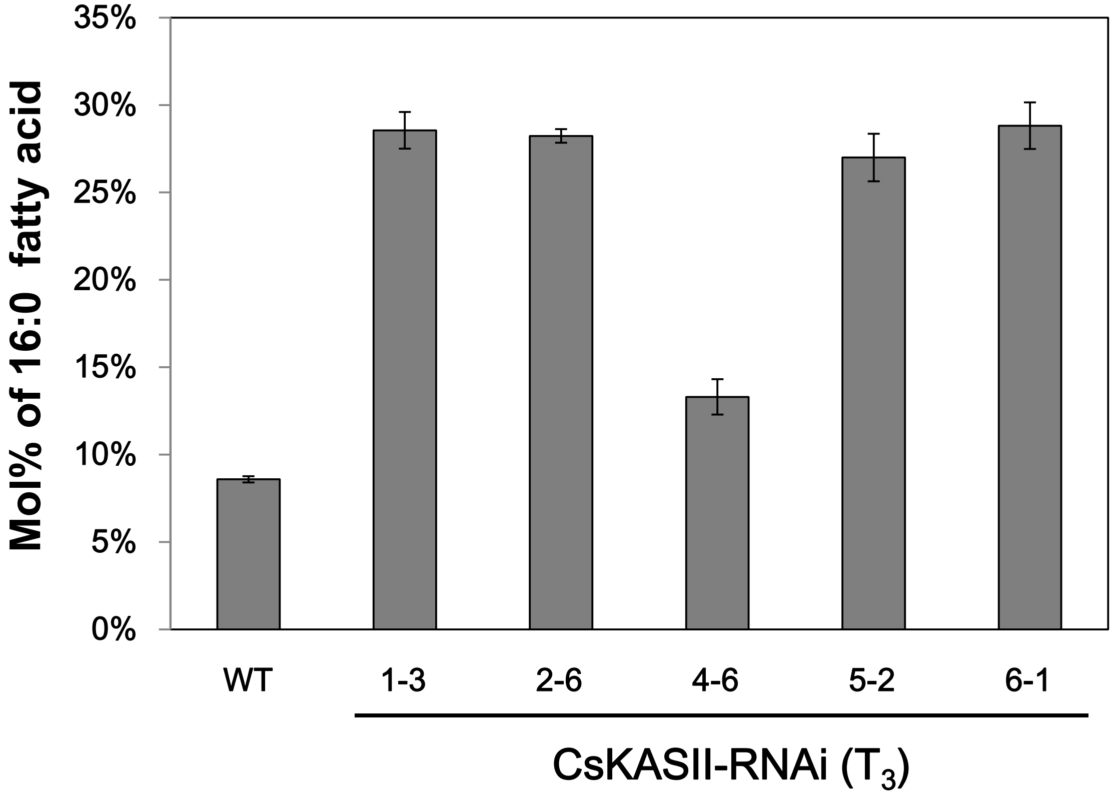

Figure S2. 16:0 Fatty acid composition of *CsKASII*-RNAi TAG.
The data represent averages of five biological replicates ± SD.

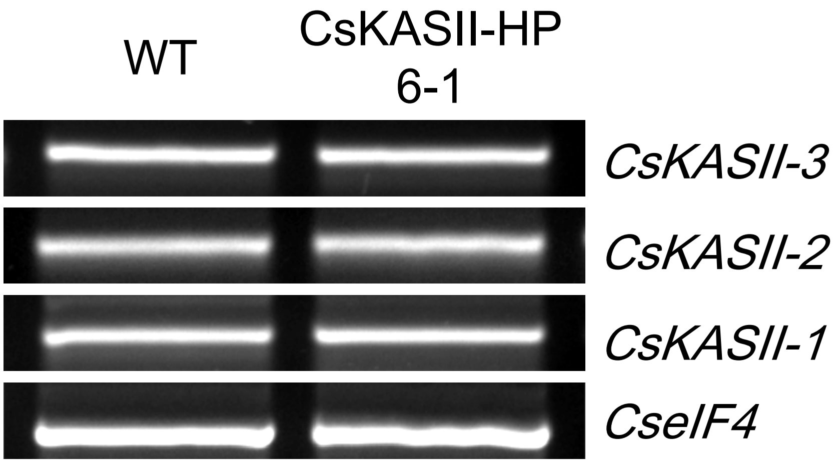

Figure S3. Transcript level of CsKASII-RNAi in developing seeds

Total RNA was isolated from 20 day-old developing seeds of Camelina and converted into cDNAs for RT-PCR analyses. Camelina *eIF4-a1* gene was used as an internal control for RT-PCR.

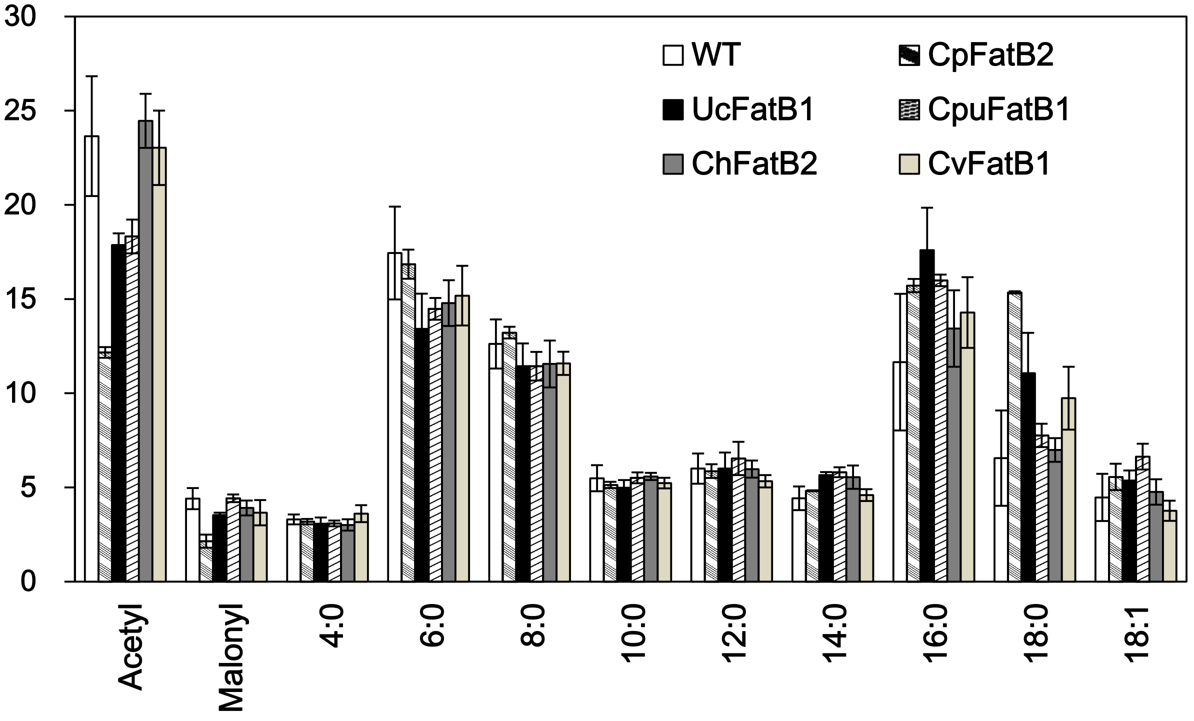

Figure S4. Acyl-ACPs analysis

Acyl-ACPs in in 10 DAF developing seeds from Camelina. The data represents means ± SD of five biological replicates.
